# Association Between Inferior Turbinate Hypertrophy and Extraesophageal Reflux

**DOI:** 10.1001/jamaoto.2022.1638

**Published:** 2022-06-30

**Authors:** Karol Zeleník, Zuzana Javorská, Renata Taimrová, Adéla Vrtková, Viktória Hránková, Miroslav Tedla, Kristína Lukáčová, Jakub Lubojacký, Martin Formánek, Pavel Komínek

**Affiliations:** 1 Department of Otorhinolaryngology and Head and Neck Surgery, University Hospital Ostrava, Ostrava, Czech Republic; 2 Department of Craniofacial Surgery, Faculty of Medicine, University of Ostrava, Ostrava, Czech Republic; 3 Department of Otolaryngology, Head and Neck Surgery, Comenius University, University Hospital, Bratislava, Slovakia; 4 Fortmedica, Prague, Czech Republic; 5 Department of Applied Mathematics, Faculty of Electrical Engineering and Computer Science, VSB-Technical University of Ostrava

## Abstract

**Importance:**

To the authors’ knowledge, no prior studies have examined the association between inferior turbinate hypertrophy (ITH) and extraesophageal reflux (EER). If EER were a cause or cofactor of ITH, antireflux treatment can be considered prior to surgical intervention.

**Objective:**

To evaluate EER presence and severity in patients with different degrees of ITH.

**Design, Setting, and Participants:**

Prospective multicentric cohort study

conducted at 3 referral centers treating patients with EER and certified for 24-hour monitoring of oropharyngeal pH. The monitoring was performed between October 2020 and October 2021.

A total of 94 adult patients with EER symptoms were recruited, 90 of whom were analyzed.

**Interventions:**

Nasal endoscopy was performed to determine the degree of ITH, according to the Camacho classification. Presence and severity of EER were examined using 24-hour monitoring of oropharyngeal pH.

**Main Outcomes and Measures:**

Primary outcomes were presence of EER according to RYAN Score, total percentage of time below pH 5.5, and total numbers of EER events below pH 5.5.

**Results:**

Of the 90 analyzed patients (median [IQR] age, 46 [33-58] years; 36 [40%] male patients), 41 had a maximum of second-degree ITH (group 1), and 49 patients had at least third-degree ITH (group 2), according to the Camacho classification. On the basis of the RYAN Score, EER was diagnosed more often in group 2 (69.4%) than in group 1 (34.1%; difference, 35.3% [95% CI, 13.5%-56.9%]). Moreover, compared with group 1, group 2 exhibited higher median total percentage of time below pH 5.5 (median [IQR], group 1: 2.1% [0.0%-9.4%], group 2: 11.2% [1.5%-15.8%]; difference, 9.1% [95% CI, 4.1%-11.8%]) and higher median total number of EER events (median [IQR], group 1: 6 [1-14] events, group 2: 14 [4-26] events; difference, 8 [95% CI, 2-15] events). Patients with proven EER demonstrated no difference in the degree of ITH between the right and left nasal cavity (Cohen *g*, −0.17 [95% CI, −0.50 to 0.30]), or between the anterior and posterior parts of the nasal cavity (Cohen *g*, −0.21 [95% CI, −0.50 to 0.17]).

**Conclusions and Relevance:**

In this cohort study, patients with a higher degree of ITH had more severe EER. A possible association between severe ITH and EER was demonstrated.

## Key Points

**Question:**

What is the association between inferior turbinate hypertrophy (ITH) and extraesophageal reflux (EER)?

**Findings:**

In this cohort study of 90 patients, 41 with Camacho second-degree ITH or lower and 49 with more severe third-degree ITH or higher, 24-hour monitoring of oropharyngeal pH revealed more patients with EER and EER of longer duration in the more severe ITH group.

**Meaning:**

A possible association between severe ITH and EER was reported.

## Teaser

This cohort study examines the association between inferior turbinate hypertrophy and extraesophageal reflux in patients at 3 referral centers.

## Introduction

Inferior turbinate hypertrophy (ITH), a frequent problem encountered in ear, nose, and throat practice, is associated with obstruction of nasal breathing and several health risks leading to mouth breathing, which is nonphysiological. Air inhaled through the mouth is not filtered, warmed, or humidified. This leads to more frequent respiratory infections, drying of the airways, and burning in the throat and can contribute to snoring and sleep apnea. Nasal obstruction results in a significantly reduced quality of life.^1,2,3,4,5^ Conservative treatment with topically applied corticosteroids is often ineffective, thus necessitating surgical reduction of the lower turbinates under local or general anesthesia. This operation is another cause of discomfort for the patient, carries risks, and should thus only be performed after the failure of conservative treatment.^2^

Extraesophageal reflux (EER) is considered a possible factor contributing to multifactorial pathogenesis of nasal disorders. Previous investigations of the role of EER in chronic rhinosinusitis, especially in difficult-to-treat conditions, indicate that EER is a likely cofactor.^6,7,8,9,10^ Moreover, EER seems to play a role in the pathogenesis of chronic otitis media with effusion and other chronic ear problems.^11,12,13^ However, to our knowledge, the association between ITH and EER has not yet been studied. If EER contributes to ITH formation, then EER treatment could be another nonsurgical therapeutic approach for patients with ITH. In the present study, we aimed to elucidate a possible association by examining EER severity using 24-hour monitoring of oropharyngeal pH in patients with varying degrees of ITH.

## Methods

This prospective cohort study was performed in accordance with the Declaration of Helsinki, the requirements of good clinical practice, and all applicable regulatory requirements. The study was approved by the institutional review board of University Hospital Ostrava and registered at ClinicalTrials.gov (NCT04581174). Written informed consent was obtained from all participants before any procedure was initiated. The study followed the Strengthening the Reporting of Observational Studies in Epidemiology (STROBE) reporting guideline.

### Patient Selection, Inclusion and Exclusion Criteria, and Questionnaires

For this study, we prospectively recruited adult patients with typical symptoms and signs of EER (most often chronic cough, chronic throat cleaning, globus pharyngeus, sore throat, dysphonia, reflux laryngitis, and difficulty in swallowing), who were scheduled for 24-hour monitoring of oropharyngeal pH between October 2020 and October 2021, at 3 referral centers treating patients with EER and certified for 24-hour monitoring of oropharyngeal pH. Enrollment of patients was consecutive. Exclusion criteria were as follows: chronic rhinosinusitis with polyps, having had an acute upper respiratory tract infection in the past 8 weeks, previous surgery in the nasal cavity and nasopharynx, history of head and neck cancer, and refusal to stop antireflux medication for the study. Enrolled patients were asked to complete a questionnaire about their personal and demographic characteristics, smoking, and medical history; the Reflux Symptom Index, in which patients rate the severity of EER symptoms from 0 to 5; and the Sino-Nasal Outcome Test (SNOT-22) questionnaire, in which patients rate symptoms and social and emotional consequences of their nasal disorders from of 0 to 5.^14,15^

### Endoscopy of the Nasal Cavity

To determine the degree of ITH, endoscopy of the nasal cavity was performed. Degree of ITH was assessed in 4 nasal cavity areas: right front, right back, left front, and left back. All 4 areas were scored for degree of ITH according to the Camacho classification: grade 1, 0% to 25%; grade 2, 26% to 50%; grade 3, 51% to 75%; and grade 4, 76% to 100% obstruction of total airway space.^16,17^

### 24-Hour Monitoring of Oropharyngeal pH

We conducted 24-hour monitoring of oropharyngeal pH using the Restech system (Respiratory Technology Corporation).^18^ Patients who were receiving long-term treatment with antireflux medication were asked to stop taking this medication before the study, as follows: proton pump inhibitor therapy was stopped for 1 week, H_2_ blockers were stopped for 48 hours, and drugs containing calcium carbonate were stopped for 1 day before the study. Omission of antireflux medication before pH monitoring at given time intervals was standardized and necessary to prevent bias in the test result. Otherwise, all patients were asked to maintain their normal daily activities. The catheter was calibrated right before examination and inserted through the more spacious nasal cavity until the flashing light-emitting diode was seen in the back of the patient’s throat. The catheter was then pulled back and positioned such that the flashing light was behind the soft palate. The catheter was secured to the patient’s face, passed over the ear, and secured to the neck. The transmitter at the end of the catheter was either taped to the skin or attached to the patient’s clothing using a clip-on case. A data recorder was attached to the patient’s belt. Patients were asked not to shower during the recording period and were instructed to record, directly to the device and manually to a diary, the time they spent eating and drinking and their position (upright or supine) during the 24-hour period. In the event of any discrepancy, periods logged in the device were modified according to the diary. Time spent eating and drinking was excluded from the analyses of pharyngeal pH recordings. The RYAN Score is an automatically calculated value from 3 components, namely the number of reflux episodes, the duration of the longest reflux episode, and the percentage of time spent below the defined pH threshold of 5.5 in the upright or 5.0 in the supine position. Patients with pathological composite RYAN Scores in the vertical position (higher than 9.4) or horizontal position (higher than 6.8) were classified as having pathological EER. We also assessed the total number of EER episodes below 5.5 and total percentage of time below pH 5.5.

### Statistical Analysis

Patients were divided into 2 study groups on the basis of ITH severity, according to the Camacho classification. Group 1 comprised patients who had a maximum of second-degree ITH in all 4 examined nasal areas, and group 2 included patients with at least third-degree ITH in at least 1 of the 4 examined nasal areas. We compared these 2 groups in terms of demographic parameters, medical history, and EER presence and severity. Demographic parameters were expressed as the median and IQR, absolute frequency, or relative frequency (%). The between-group differences in analyzed numerical parameters are presented as the differences in group medians with 2-sided 95% CIs, obtained using the bootstrap procedure. In 2 × 2 contingency tables, the between-group differences were presented with the differences in group proportions (%) with 2-sided 95% CIs, obtained using Pearson’s χ^2^ test. The Cramer *V* with its CI was used to evaluate the association in larger contingency tables, and Cohen *g* and its CI was used as a measure of an effect size for paired contingency tables. The significance level was set to .05. Statistical analyses were performed using R statistical software, version 4.1.2 (R Project for Statistical Computing).

## Results

A total of 94 patients were recruited for the study, and 4 (4%) were excluded from statistical analysis because of intolerance of catheter (1 patient) or technical problems during 24-hour monitoring of oropharyngeal pH (3 patients). Among the remaining 90 patients, 36 (40%) were male patients, and 54 (60%) were female patients. The median (IQR) age was 46 (33-58) years. A total of 41 patients (46%) were in group 1, and 49 patients (54%) were in group 2. These groups did not differ in demographic data or smoking history (Table 1). We also found no between-group difference in the presence of heartburn or allergic rhinitis and in the values of the Reflux Symptom Index and SNOT-22 (Table 2).

**Table 1.** Participants’ Sex, Age, BMI, and Smoking History

Characteristic	Median (IQR) or No. (%)			Effect size (95% CI)
	Total (n = 90)	Group 1 (n = 41)^a^	Group 2 (n = 49)^a^	
Sex				
Female	54 (60)	25 (61)	29 (59)	−1.8 (−23.9 to 20.3)^b^
Male	36 (40)	16 (39)	20 (41)	1.8 (−20.3 to 23.9)^b^
Age, y	46 (33 to 58)	44 (29 to 58)	49 (36 to 58)	5 (−3 to 12)^c^
BMI	25.6 (23.0 to 28.7)	24.5 (22.4 to 28.7)	25.8 (23.1 to 28.7)	1.3 (−1.2 to 2.5)^c^
Smoking, yes	19 (21)	7 (17)	12 (25)	7.4 (−11.5 to 26.3)^b^

Abbreviation: BMI, body mass index, calculated as weight in kilograms divided by height in meters squared.

^a^ Group 1 had a maximum of Camacho second-degree inferior turbinate hypertrophy; group 2, at least third-degree inferior turbinate hypertrophy.

^b^ The difference between group proportions (in %, group 2 − group 1).

^c^ The difference between group medians (group 2 − group 1).

**Table 2.** Participants’ Medical History, Reflux Symptom Index (RSI), and Sino-Nasal Outcome Test (SNOT-22) Questionnaire

Characteristic	Median (IQR) or No. (%)			Effect size (95% CI)
	Total (n = 90)	Group 1 (n = 41)^a^	Group 2 (n = 49)^a^	
Heartburn				0.06 (0.02 to 0.32)^b^
More often than once a week	19 (21)	8 (20)	11 (22)	
Once a week or less	22 (24)	11 (27)	11 (22)	
Never	49 (55)	22 (54)	27 (55)	
Presence of allergic rhinitis	35 (39)	13 (32)	22 (45)	13.2 (−9.0 to 35.4)^c^
RSI (absolute value comparison)	13 (9 to 20)	15 (9 to 21)	12 (9 to 19)	−3 (−4 to 2)^d^
RSI (RSI ≤ 12)	41 (46)	16 (39)	25 (51)	12.0 (−10.7 to 34.7)^c^
SNOT-22	22 (11 to 33)	20 (11 to 34)	22 (12 to 32)	2 (−6 to 6)^d^

^a^ Group 1 had a maximum of Camacho second-degree inferior turbinate hypertrophy; group 2, at least third-degree inferior turbinate hypertrophy.

^b^ Cramer *V*.

^c^ The difference between group proportions (in %, group 2 − group 1).

^d^ The difference between group medians (group 2 − group 1).

Based on the RYAN Score, EER was diagnosed more often in group 2 (69.4%) than in group 1 (34.1%; difference, 35.3% [95% CI, 13.5%-56.9%]). Compared with group 1, group 2 also demonstrated higher median total percentage of time below pH 5.5 (median [IQR], group 1: 2.1% [0.0%-9.4%], group 2: 11.2% [1.5%-15.8%]; difference, 9.1% [95% CI, 4.1%-11.8%]) and higher median total number of EER events (median [IQR], group 1: 6 [1-14] events, group 2: 14 [4-26] events; difference, 8 [95% CI, 2**-**15] events) (Table 3). Among the 48 patients with proven EER, we found no difference in the degree of ITH between the right and left nasal cavity (Cohen *g*, −0.17 [95% CI, −0.50 to 0.30]) or in the degree of ITH between the anterior and posterior parts of the nasal cavity (Cohen *g*, −0.21 [95% CI, −0.50 to 0.17]) (Table 4).

**Table 3.** Extraesophageal Reflux (EER) Diagnostic Parameters in Both Study Groups

Outcome	Median (IQR) or No. (%)			Effect size (95% CI)
	Total (n = 90)	Group 1 (n = 41)^a^	Group 2 (n = 49)^a^	
EER according to RYAN Score	48 (53)	14 (34)	34 (69)	35.3 (13.5-56.9)^b^
Total % time below pH 5.5	5.2 (0.3-14.5)	2.1 (0.0-9.4)	11.2 (1.5-15.8)	9.1 (4.1-11.8)^c^
Total No. of events	11 (2-22)	6 (1-14)	14 (4-26)	8 (2-15)^c^

^a^ Group 1 had a maximum of Camacho second-degree inferior turbinate hypertrophy; group 2, at least third-degree inferior turbinate hypertrophy.

^b^ The difference between group proportions (in %, group 2 − group 1).

^c^ The difference between group medians (group 2 − group 1).

**Table 4.** Participants’ Turbinate Grades at 4 Examined Sites (Right Anterior, Right Posterior, Left Anterior, Left Posterior)^a^

Characteristic	Right Camacho ≤ 2	Right Camacho ≥ 3	Cohen *g* (95% CI)
Left Camacho ≤ 2	14	2	−0.17 (−0.50 to 0.30)
Left Camacho ≥ 3	4	28	
	**Posterior Camacho ≤ 2**	**Posterior Camacho ≥ 3**	
Anterior Camacho ≤ 2	14	2	−0.21 (−0.50 to 0.17)
Anterior Camacho ≥ 3	5	27	

^a^ The values represent the absolute frequencies.

## Discussion

In this cohort study, the results demonstrated that there is a possible association between ITH and EER. Using 24-hour monitoring of oropharyngeal pH in our study population, EER was detected more often in patients with more severe ITH. Notably, we did not find differences in other factors that may contribute to ITH, namely smoking and allergies. The results of our study are in concordance with a few recent studies focused on EER, rhinitis, and nasal patency. Dagli et al^19^ reported findings that support the role of EER in nasal patency deterioration and reported that EER was associated with increased nasal resistance and nasal congestion. Moreover, they found that EER treatment was associated with improved subjective (Nasal Obstruction Symptom Evaluation score) and objective nasal findings (nasal patency determined by rhinomanometry).^19^ Additionally, Finocchio et al^20^ demonstrated that gastritis and gastroesophageal reflux disease were associated with nasal disturbances and that patients with nonallergic rhinitis and sinusitis had a greater relative risk ratio than those with allergic rhinitis.

Surprisingly, prior studies have included only limited investigations of EER as a potential contributing factor of ITH. Yet it is possible that EER likely plays some role in the pathogenesis of nasal and ear chronic inflammatory conditions, such as chronic rhinosinusitis, recurrent otitis media, chronic otitis media with effusion, and others.^6,7,8,9,10,11,12,13^ Notably, the European Position Paper on Rhinosinusitis and Nasal Polyps supports further studies to elucidate the association between EER and chronic nasal inflammatory conditions.^4^

Extraesophageal reflux (also called laryngopharyngeal reflux) is currently defined as an inflammatory condition of the upper aerodigestive tract tissues associated with the direct and indirect effects of gastric or duodenal content reflux, inducing morphological changes in the upper aerodigestive tract.^13^ Studies over the past decade have examined the association between EER and chronic rhinosinusitis but not simple turbinate hypertrophy. A potential association among gastroesophageal reflux disease, EER (laryngopharyngeal reflux), and chronic rhinosinusitis has long been suspected in both adults and children. Indeed, Jecker et al^8^ demonstrated that patients with recurrent chronic rhinosinusitis have more laryngopharyngeal reflux complaints, increased esophageal reflux events, and impaired pH-metry findings compared with healthy volunteers. Additional studies have found more hypopharyngeal or nasopharyngeal reflux episodes in patients with refractory chronic rhinosinusitis and/or early recurrence of nasal polyps after functional endoscopic sinus surgery compared with patients without recurrence.^6,7^ Moreover, Lechien et al^10^ conducted a 10-year follow-up of patients with chronic rhinosinusitis and demonstrated positive associations among the severity of gastroesophageal reflux disease complaints, SNOT-20 scores, and late recurrence of chronic rhinosinusitis requiring functional endoscopic sinus surgery. Overall, the available data suggest an association between EER and severe chronic rhinosinusitis.^6,7,8,9,10^

There is presently no reference standard for EER examination.^13^ Analysis of 24-hour esophageal pH impedance seems to provide the most accurate data for determining EER presence, type, and severity. Data from 24-hour esophageal pH–impedance analysis provide important information about reflux episodes in the esophagus, hypopharynx, and near the larynx. However, it cannot determine how many reflux episodes reach the nasopharynx and nasal cavity. Therefore, determination of reflux in the nasopharynx and nasal cavity is more complicated and less standardized than in the hypopharynx. To date, the most suitable tool for that purpose seems to be 24-hour monitoring of oropharyngeal pH (Restech system) with a probe positioned at the level of the nasopharynx.^18^ In our study, we used this method for detecting and quantifying reflux episodes that reached the nasopharynx and nasal cavity. The detailed results of our study demonstrate that the degree of ITH did not differ between the right and left nasal cavity or between the anterior and posterior parts of the nasal cavity among patients with proven EER. Probable interpretation is that EER reaches both posterior sides of the nasal cavity and causes secondary inflammation of the entire turbinate and nasal tissue.

Another method for determining the amount of reflux that reaches the nasal cavity is the detection of pepsin in nasal lavage fluid. A few studies have examined this topic,^21,22,23^ concluding that pepsin is commonly detected in patients with chronic rhinosinusitis, particularly in those with medically and surgically refractory chronic rhinosinusitis. There remains a need for larger multicentric studies to validate pepsin detection in nasal lavage fluid as a tool for the diagnosis of EER in the nasal cavity.

In summary, EER seems to be a currently underestimated factor associated with ITH. It is not clear how EER causes chronic inflammatory changes in the nasal mucosa, but low pH and pepsin seem to play a role. Low pH leads to increased junctional permeability through the disruption of protein bridge formation with cell-to-cell adhesion molecules, such as E-cadherin.^24^ Moreover, even under neutral pH, pepsin can increase the expression of the heat shock protein HSP70 in human nasal epithelial cells by activating the JNK/MAPK signaling pathway.^25^ This appears to be 1 mechanism through which EER can contribute to ITH and chronic rhinosinusitis.^26^

### Limitations

Our study had several limitations. First, because of the limited number of included patients, the results should be interpreted cautiously. Second, the Camacho staging of the degree of ITH is subjective. However, this limitation also applies for all questionnaires and all visual examinations of the nasal cavity. The Camacho classification is a validated grading scale with high intrarater and interrater reliability, so we considered it useful for our research. Moreover, objective examination using rhinomanometry, acoustic rhinometry, and computed tomography is also influenced by the nasal cycle. Third, to confirm the role of EER in the pathogenesis of ITH, there remains a need for a robust study on the effects of an antireflux diet and medical therapy on ITH reduction.

## Conclusions

In this cohort study, patients with a higher degree of ITH were more commonly diagnosed with more severe EER using 24-hour monitoring of oropharyngeal pH. A possible association between severe ITH and EER was reported.
